# The Association between TGF-β1 Polymorphisms and Radiation Pneumonia in Lung Cancer Patients Treated with Definitive Radiotherapy: A Meta-Analysis

**DOI:** 10.1371/journal.pone.0091100

**Published:** 2014-03-18

**Authors:** Jiazhuo He, Lei Deng, Feifei Na, Jianxin Xue, Hui Gao, You Lu

**Affiliations:** Department of Thoracic Oncology, Cancer Center, West China Hospital, Sichuan University, Chengdu, Sichuan, PR China; University of Birmingham, United Kingdom

## Abstract

**Background:**

Previous studies investigating the association between TGF-β1 polymorphisms and Radiation Pneumonia (RP) risk have provided inconsistent results. The aim of our study was to assess the association between the TGF-β1 genes C509T, G915C and T869C polymorphisms and risk of RP in lung cancer patients treated with definitive radiotherapy.

**Methods:**

Two investigators independently searched the Medline, Embase, CNKI, and Chinese Biomedicine Databases for studies published before September 2013. Summary odds ratios (ORs) and 95% confidence intervals (CIs) for TGF-β1 polymorphisms and RP were calculated in a fixed-effects model or a random-effects model when appropriate.

**Results:**

Ultimately, each 7 studies were found to be eligible for meta-analyses of C509T, G915C and T869C, respectively. Our analysis suggested that the variant genotypes of T869C were associated with a significantly increased RP risk in dominant model (OR = 0.59, 95% CI = 0.45–0.79) and CT vs. TT model (OR = 0.47, 95% CI = 0.32–0.69). In the subgroup analyses by ethnicity/country, a significantly increased risk was observed among Caucasians. For C509T and G915C polymorphism, no obvious associations were found for all genetic models.

**Conclusion:**

This meta-analysis suggests that T869C polymorphism of TGF-β1 may be associated with RP risk only in Caucasians, and there may be no association between C509T and G915C polymorphism and RP risk.

## Introduction

Radiation Pneumonia (RP) is an inflammation of the lungs due to radiation therapy. This side effect of radiation therapy occurs in 5 to 15% of people who go through radiation therapy for lung cancer. Radiation Pneumonia (RP) is a dose-limiting factor for radiotherapy and have a major influence on patient;s prognosis and live quality. Because current parameters for predicting RP are very limited, there is a demand for the possibility of developing novel parameters. Although many factors can influence the severity of reaction to radiotherapy, a large part of interpatient variability attribute to the individual differences in radiosensitivity, which was assumed to be determined by genetic variations among patients [1]. Recently, studies have focused on the association of common polymorphic variations in candidate genes and individual differences in radiosensitivity [2].

Transforming growth factor beta 1(TGF-β1) gene controls proliferation, cellular differentiation, and other functions in most cells. The TGF-β1 signaling pathway is involved in many cellular processes in both the adult organism and the developing embryo including cell growth, cell differentiation, apoptosis, cellular homeostasis and other cellular functions. TGF-β1 is one of the most extensively studied cytokines in the development of tissue fibrosis in response to irradiation and TGFβ1 signaling is an important modulator of the inflammatory response. Several recent studies have investigated plasma levels of TGFβ1 as a predictor for RT-induced lung injury. Animal and human studies have demonstrated that TGF-β1 is a major regulator of radiation-induced lung injury [3]–[7]. Administration of anti-TGF-β1 antibodies can decrease the inflammatory response and reduce TGF-β1 activation several weeks after radiotherapy, further suggesting that targeting the TGFβ pathway may be a useful strategy to prevent RT-induced lung injury [8]. A decrease in plasma TGF-β1 concentration to that of less than the pretreatment value during radiotherapy in patients without pulmonary complications supports the use of TGF-β1 as a predictive biomarker [5]. However, improper handling of blood samples or inadequate centrifugation conditions can falsely increase the level of circulating TGF-β1, which seriously limits this approach for risk prediction.

Radiogenomics with genotyping analysis of SNPs in TGFβ1 and TGFβ1 pathway genes may allow the identification of genotypes prone to RP. Previous studies have demonstrated that the TGFβ1 alleles are mainly clustered into three phylogenetic groups based on the common functional SNPs C509T, G915C and T869C, suggesting three phenotypic groups [9]. The C509T, G915C and T869C polymorphisms of TGF-β1 lead to amino acid substitutions in TGF-β1, which may alter TGF-β1 function. This change in protein biochemistry leads to the supposition that variant alleles may diminish repair kinetics, thereby influencing susceptibility to adverse health effects [10]. As the functions of the TGF-β1 C509T, G915C and T869C genes make them candidates for association with RP, a number of case–control studies were conducted to investigate the association between TGF-β1 C509T, G915C and T869C polymorphisms and risk of RP or other normal tissue complications [11]–[19]. But these studies reported conflicting results. Different methodologies have been used, but, in particular, some of the studies used a small sample size and it is therefore not surprising that there has been a lack of replication in the various studies. By using all the available published data to increase the statistical power, it was hypothesized that a meta-analysis might allow plausible candidate genes to be excluded and causative genes to be identified with reliability. We have therefore taken a meta-analysis in which all the published case-control studies are processed to confirm whether the C509T, G915C and T869C polymorphism of TGF-β1 gene promoter increased the risk of RP.

## Materials and Methods

### Literature search strategy

The PubMed, Embase, HuGE Navigator (http://www.hugenavigator.net) and China National Knowledge Infrastructure(CNKI) we researched to retrieve all papers available from inception to Nov 5, 2013, using both free words and index terms specific to each search platform (MeSH in PubMed and Emtree in Embase). The search strategies were based on combinations of the key words (‘TGF-β1’ or ‘TGFbeta’) and (‘polymorphism’ or ‘genotype’ or ‘genetic’) and (‘Radiation Pneumonia’ or ‘Radiation Pneumonitis’ or ‘Radiation Induced Lung Toxicity’). The references in the studies were reviewed to identify additional studies that were not indexed by PubMed, Embase, HuGE Navigator, and CNKI. All published papers in English language and Chinese language with available full text matching the eligible criteria were retrieved. In addition, we also checked the references of relevant reviews and eligible articles that our search retrieved. If more than one article were published by the same author using the same case series, we selected the study where the most individuals were investigated.

### Selection criteria and identification of studies

For inclusion in this meta-analysis, the identified articles had to provide information on the following: (i) Exploration of TGF-β1 C509T, G915C or T869C polymorphisms and risk of RP, (ii) using a case–control design; (iii) sufficient data for examining an odds ratio (OR) with 95% confidence interval (CI); (iv) the most recent and/or the largest study with extractable data should be included concerning studies with overlapping patients and the controls. Major reasons for the exclusion of studies were as follows: (i) duplicate data, (ii) abstract, comment, review and editorial and (iii) no sufficient data were reported.

### Data extraction

According to the inclusion and exclusion criteria, extraction from each study was conducted independently by two authors (J.H and F.N.) and the consensus was achieved for all the data. The following information was extracted: the first author's name, year of publication, country, ethnicity, numbers of cases/patients and controls, and distribution of genotypes in the case and control groups. For studies with inadequate information, authors of those studies were contacted for further information by E-mail if possible.

### Statistical analysis

Meta-analysis was performed and reported as described previously. Crude ORs with 95% CIs were computed to assess the strength of the correlation between the TGF-β1 polymorphisms and the susceptibility to RP. The pooled ORs were performed for the dominant model (aa+Aa vs. AA) and recessive model (aa vs. Aa+AA). Moreover, the pooled estimates were also calculated for the pair-wise comparisons (allele Aa vs. AA, and allele aa vs. AA). The above-mentioned A and a represented the major and the minor alleles, respectively. Taking consideration of possible between-study heterogeneity, a statistical test for heterogeneity was performed by the x^2^ test or Fisher exact test when appropriate. P<0.10 or I^2^>50% indicated an obvious of the between-study heterogeneity, and OR (95% CI) was calculated by the random-effects model using the DerSimonian and Laird method; otherwise, the fixed-effects model was used by the Mantel-Haenszel method. Subgroup analyses were mainly conducted on ethnicity (Asian, Caucasian),which were used to explore and explain the heterogeneity between the different studies. Sensitivity analyses were performed on stability of the results, namely, one case-control study omitted each time to reflect the influence of the individual data set on the pooled OR. Asymmetry of the funnel plot indicated the possible publication bias. All analyses were performed using the RevMan 5.2 program (Cochrane Library, UK) or the STATA package version 13.0 program (Stata Corporation, College Station, Texas, USA). All P values were two-sided. To ensure the reliability of data, two reviewers (J.H and F.N.) independently performed the data analysis using the statistics programs for the same results.

## Results

### Literature search and study selection

**Figure 1:**
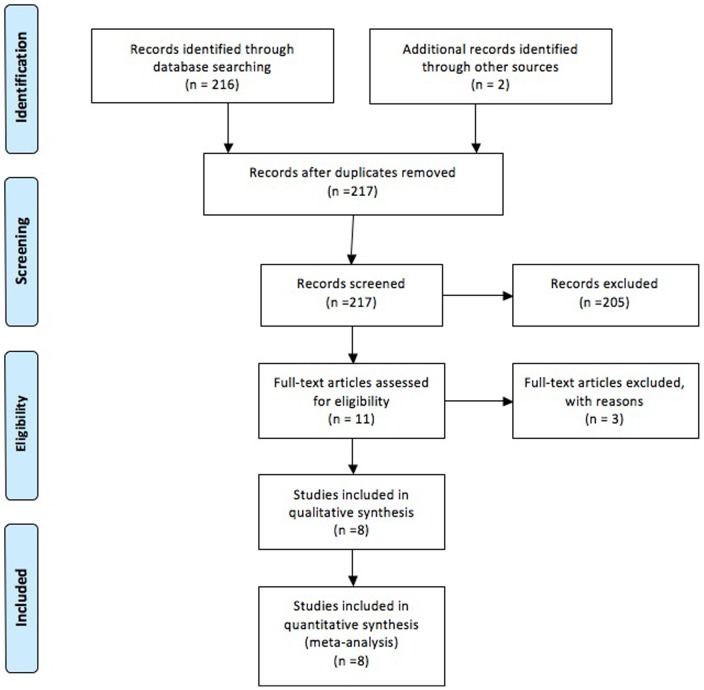
The literature search and study selection procedures.

217 studies were identified by initial search. Through screening the title and reading the abstract and the entire article, 8 eligible articles (6 in English and 2 in Chinese) were included based on the search criteria, for RP susceptibility related to the TGF-β1 C509T, G915C or T869C polymorphisms. The literature search and study selection procedures are shown in Figure 1. Study characteristics were summarized in Table 1. Studies were conducted in China, Belgium, Argentina, US and Saudi Arabia. The controls were lung cancer patients with comparable clinical characteristics who did not develop RP.

**Table 1:** Characteristics of the included case-control studies on the TGF-β1 polymorphisms and Radiation Pneumonia (RP) risk.

Gene	Author	Year	Ethnicity	Country	Sample Size		Case						Control					
					Case	Control	C	T	CC	CT	TT	CT+TT	C	T	CC	CT	TT	CT+TT
C-509T rs1800469	Niu	2012	Asian	China	46	121	39	53	8	23	15	38	109	133	21	67	33	100
	Tucker	2012	Caucasian	US	28	115	49	7	21	7	0	7	175	51	66	43	4	47
	Voets	2012	Caucasian	Belgium	59	150	88	30	31	26	2	28	209	91	70	69	11	80
	Wang	2010	Asian	China	63	107			16			47			28			79
	Wang	2011	Asian	China	38	96	40	36	13	14	11	25	88	104	30	28	38	66
	Yuan	2009	Caucasian	US	74	89			47			27			53			36
	Zhang	2008	Asian	China	29	141	35	23	9	17	3	20	140	142	35	70	36	106
					337	819	251	149	145	87	31	192	721	521	303	277	122	514

Gene	Author	Year	Ethnicity	Country	Sample Size		Case						Control					
					Case	Control	G	C	GG	GC	CC	GC+CC	G	C	GG	GC	CC	GC+CC
G915C rs1800471	Niu	2012	Asian	China	46	121	92	0	46	0	0	0	240	2	120	1	0	1
	Tucker	2012	Caucasian	US	28	113	54	2	26	2	0	2	208	18	98	12	3	15
	Voets	2012	Caucasian	Belgium	59	150	109	10	49	10	0	10	287	13	137	13	0	13
	Wang	2010	Asian	China	179	0	358	0	179	0	0	0	0	0	0	0	0	0
	Wang	2011	Asian	China	38	96	76		38			0	192		96			
	Yuan	2009	Caucasian	US	74	89			33			3			111			17
	Zhang	2008	Asian	China	29	141	35	23	14	7	8	15	164	120	46	72	24	96

Gene	Author	Year	Ethnicity	Country	Sample Size		Case						Control					
					Case	Control	T	C	TT	TC	CC	TC+CC	T	C	TT	TC	CC	TC+CC
T869C rs1982073	Niu	2012	Asian	China	46	121	38	54	8	22	16	40	107	135	20	67	34	101
	Tucker	2012	Caucasian	US	28	113	42	14	16	10	2	12	129	97	30	69	14	83
	Voets	2012	Caucasian	Belgium	59	150	88	30	36	16	7	23	213	87	77	59	14	73
	Wang	2010	Asian	China	64	115			18			46			36			79
	Wang	2011	Asian	China	38	96	46	30	17	12	9	21	90	92	23	44	29	73
	Yuan	2009	Caucasian	US	74	89			17			18			35			93
	Alsbeih	2010	Arabian	Saudi Arabia	30	30	40	20	15	10	5	15	27	33	8	11	11	22

### Quantitative synthesis

#### Association between the TGF-β1 T869C polymorphism and risk of RP

**Figure 2:**
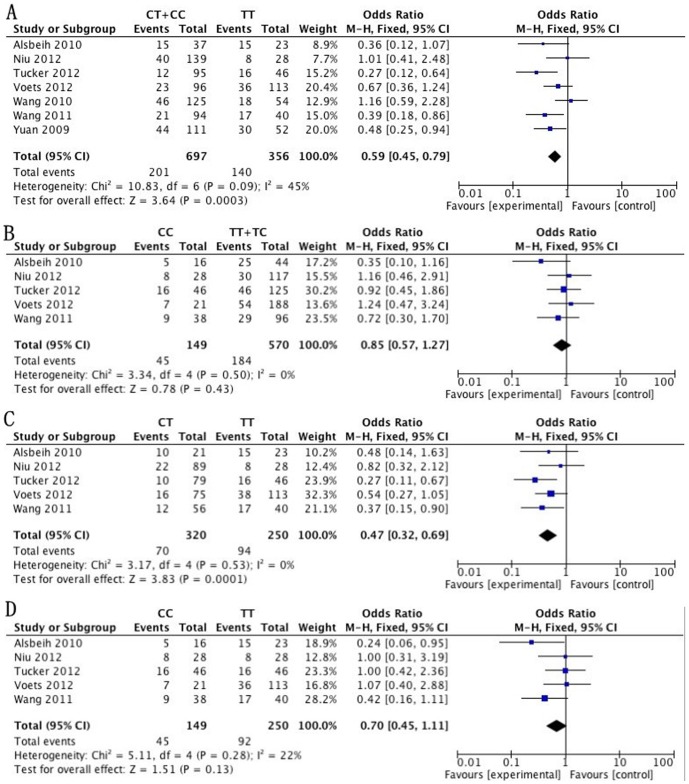
Forrest plot of association between the TGF-β1 T869C polymorphism and risk of RP. (A)Meta-analysis in a fix effects model for dominant model. (B) Meta-analysis in a fix effects model for recessive model. (C) Meta-analysis in a fix effects model for CT vs. CC. (D) Meta-analysis in a fix effects model for CC vs. TT.

**Figure 3:**
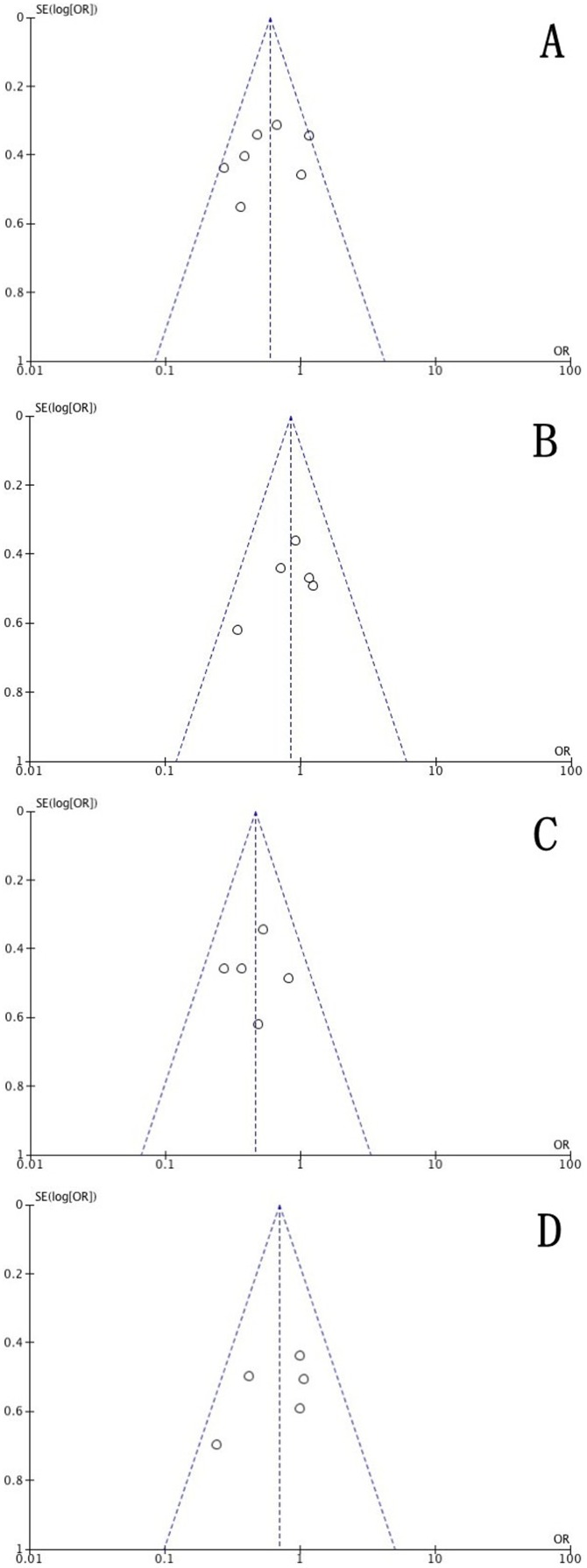
Funnel plot analysis on the detection of the publication bias for the T869C polymorphism. (A)Meta-analysis in a fix effects model for dominant model. (B) Meta-analysis in a fix effects model for recessive model. (C) Meta-analysis in a fix effects model for CT vs. CC. (D) Meta-analysis in a fix effects model for CC vs. TT. Each point represents an individual study for the indicated association.

7 case-control studies with 341 cases and 712 controls were included for association between TGF-β1 T869C polymorphism and RP risk. The evaluations of the association of TGF-β1 T869C polymorphism with RP risk are shown in Table 2. The results of the combined analyses showed that TGF-β1 T869C polymorphism was associated with lower RP risk for dominant model (OR = 0.59, 95% CI = 0.45–0.79) and CT vs. TT model (OR = 0.47, 95% CI = 0.32–0.69). However, TGF-β1 T869C polymorphism was not associated with RP risk for recessive model (OR = 0.85, 95% CI = 0.57–1.27) and CC vs. TT model (OR = 0.70, 95% CI = 0.45–1.11) (Table 2). In the subgroup analyses by ethnicity/country, a significant association of TGF-β1 T869C with lower RP risk was observed among Caucasian (dominant model: OR = 0.48, 95% CI = 0.29–0.78). However, among Asian, no significantly increased risk was observed (dominant model: OR = 0.80, 95% CI = 0.32–1.25). The heterogeneity was significant in the dominant model for Asian subgroup (I^2^ = 57%, P = 0.10) and the random-effects model was performed. However, there was no significant heterogeneity for the comparison of other genetic models (P>0.1) and the fixed effects method was performed for our investigation. No obvious publication bias was detected according to the funnel plots for the T869C polymorphism in all the genetic models (Figure 2, 3).

**Table 2:** Pooled Analysis on Association between the T869C polymorphism and the RP risk.

Genetic Model	Number of study	Sample size		Analysis Model	I^2^	Test of Association(95%CI)	
		case	control			P	OR
Dominant model	7	341	712	F	45	0.00	0.59 [0.45, 0.79]
Recessive model	5	229	490	F	0	0.43	0.85 [0.57, 1.27]
CT vs. TT	5	164	406	F	0	0.00	0.47 [0.32, 0.69]
CC vs. TT	5	137	262	F	22	0.13	0.70 [0.45, 1.11]
Ethnicity							
Asian							
Dominant model	3	150	330	R	57	0.33	0.80 [0.52, 1.25]
Caucasian							
Dominant model	3	161	352	F	30	0.00	0.48 [0.29, 0.78]

Dominant model: TC+CC vs.TT; Recessive model: CC vs. TT+TC; R, Random-effects model; F, fixed-effects model;

#### Association between the TGF-β1 C509T polymorphism and risk of RP

As for the C509T polymorphism, 7 studies with 337 cases and 817 controls were included eventually. No association was found between the polymorphism and the susceptibility to RP in all the genetic models (Table 3, dominant model: OR = 0.82, 95% CI = 0.62–1.08; recessive model: OR = 0.74, 95% CI = 0.47–1.18; CT vs. CC: OR = 0.84, 95% CI = 0.58–1.22; TT vs. CC: OR = 0.63, 95% CI = 0.36–1.08). The subgroup analysis of the C509T polymorphisms in the ethnicity group revealed that no association was found between the C677T polymorphism and the risk of RP in either the Asian or the Caucasian populations in all the genetic models (Table 3). The heterogeneity were not significant in all the genetic models (P>0.05) and the fixed-effects model was used in the meta-analysis. No obvious publication bias was detected according to the funnel plots for the C509T polymorphism in all the genetic models.

**Table 3:** Pooled Analysis on Association between the C509T polymorphism and the RP risk.

C509T							
Genetic Model	Number of study	Sample size		Analysis Model	I^2^	Test of Association(95%CI)	
		case	control			P	OR
Dominant model	7	337	817	F	0	0.16	0.82 [0.62, 1.08]
Recessive model	5	171	600	F	5	0.21	0.74 [0.47, 1.18]
CT vs. CC	5	169	499	F	0	0.36	0.84 [0.58, 1.22]
TT vs. CC	5	113	344	F	0	0.10	0.63 [0.36, 1.08]
Ethnicity							
Asian							
Dominant model	4	176	465	F	0	0.68	0.92 [0.61, 1.38]
Recessive model	3	113	358	F	48	0.25	0.75 [0.46, 1.23]
CT vs. CC	3	84	251	F	0	0.92	1.00 [0.59, 1.70]
TT vs. CC	3	59	193	F	11	0.55	0.70 [0.39, 1.28]
Caucasian							
Dominant model	3	161	352	F	0	0.12	0.73 [0.49, 1.09]
Recessive model	2	58	242	F	0	0.60	0.69 [0.18, 2.71]
CT vs. CC	2	84	251	F	0	0.99	1.00 [0.59, 1.70]
TT vs. CC	2	54	151	F	0	0.19	0.39 [0.10, 1.57]

Dominant model: CT+TT vs. CC; Recessive model: TT vs. CC+CT; Additive model: T vs. C; R, Random-effects model; F, fixed-effects model;

#### Association between the TGF-β1 G915C polymorphism and risk of RP

As for the G915C polymorphism, 7 studies with 453 cases and 710 controls were included eventually. No association was found between the polymorphism and the risk of RP in all the genetic models (Table 4, dominant model: OR = 0.91, 95% CI = 0.57–1.46; recessive model: OR = 1.60, 95% CI = 0.67–3.80; GC vs. GG: OR = 1.07, 95% CI = 0.31–3.68; CC vs. GG: OR = 1.00, 95% CI = 0.39–2.56). The subgroup analysis of the G915C polymorphisms in the ethnicity group revealed that no association was found between the G915C polymorphism and the risk of RP in either the Asian or the Caucasian populations in the dominant models (Table 4). The heterogeneity was significant in the GC vs. GG model (I^2^ = 67%, P = 0.03) and the random-effects model was performed. However, there was no significant heterogeneity for the comparison of other genetic models (P>0.1) and the fixed effects method was performed for our investigation. No obvious publication bias was detected according to the funnel plots for the G915C polymorphism in all the genetic models.

**Table 4:** Pooled Analysis on Association between the G915C polymorphism and the RP risk.

G915C							
Genetic Model	Number of study	Sample size		Analysis Model	I^2^	Test of Association(95%CI)	
		case	control			P	OR
Dominant model	7	453	710	F	33	0.69	0.91 [0.57, 1.46]
Recessive model	5	200	593	F	0	0.29	1.60 [0.67, 3.80]
CG vs. GG	5	192	586	R	67	0.91	1.07 [0.31, 3.68]
CC vs. GG	5	322	428	F	0	1.00	1.00 [0.39, 2.56]
Ethnicity							
Asian							
Dominant model	4	292	357	F	0	0.13	0.54 [0.25, 1.19]
Caucasian							
Dominant model	3	161	353	F	34	0.52	1.21 [0.68, 2.16]

Dominant model: GC+CC vs. GG; Recessive model: CC vs. GG+GC; Additive model: C vs. G; R, Random-effects model; F, fixed-effects model;

## Discussion

In recent years, several molecular epidemiological studies have been conducted to evaluate the role of polymorphisms in the TGF-β1 on RP susceptibility in lung cancer patients; however, the results remain conflicting rather than conclusive. Three polymorphisms in TGF-β1 C509T, G915C and T869C have been frequently examined in the studies on RP susceptibility. To the best of our knowledge, this is the first systematic review that has investigated the association of TGF-β1 polymorphisms and RP susceptibility. In this meta-analysis, we found that TGF-β1 T869C polymorphism may be associated with RP susceptibility only in Caucasian, and there may be no association between C509T and G915C polymorphism and RP susceptibility.

Because the allele frequencies of polymorphisms and their effects on the RP susceptibility were diverse in the different ethnicities, we carried out subgroup analysis by ethnicity. The results of combined analyses suggested that the T869C polymorphism was associated with an increased RP susceptibility, while the TGF-β1 C509T and G915C was not associated with RP susceptibility when all the studies were pooled. It is reported that there is a wide variation in the TGF-β1 T869C C allele frequency among different ethnicities, ranging from 46.9% in an Asian population to 36.9% in a Caucasian population [14]. When stratifying by ethnicity, a significantly increased risk was observed among Caucasian for the T869C. However, no association between T869C polymorphism and RP risk was found in Asian. Studies on the association of TGF-β1 polymorphisms with RP were predominantly conducted in Asian countries; only 3 were conducted in Western countries. Thus, possible ethnic differences in the association of TGF-β1 polymorphisms with RP should be investigated further and confirmed by more future studies are conducted in Caucasian patients. These results implies that genetic factors may have played a greater role in influencing individual patient susceptibility, suggesting the possibility of using these biomarkers as predictive factors. In additional, future understanding of the combined effect of these polymorphisms on patient response to radiotherapy may shed some light on the predictive value of these genetic factors.

To some extent, several limitations of this meta-analysis should be addressed. We did not perform subgroup analysis by the pathological types of lung cancer due to limited data in primary studies. Because of different pathological types, subgroup analysis should be performed. However, some study in this meta-analysis didn't report separate genotype frequency for each pathological type of lung cancer, which prevented us to perform this subgroup analysis. Another limitation of the present study was that the sample size involved is not big enough. The number of studies and the number of subjects in the studies included in the meta-analysis were small. Additional meta-analyses with a large number of papers are necessary to validate the association in the future. Besides, part of the exposure information was still lacking in the available studies, E.g., smoking status or nutritional status. Therefore, effects of environment exposure or lifestyle on association between TGF-β1 variants and susceptibility for radiation pneumonia could not be determined by this meta-analysis.

In conclusion, the present study provides evidence that the TGF-β1 T869C polymorphism may be associated with RP risk only in Caucasian, and there may be no association between TGF-β1 C509T and G915C polymorphism and RP risk. The association between the TGF-β1 T869C polymorphisms and the susceptibility for radiation pneumonia need to be further studied.

## Supporting Information

Checklist S1 **PRISMA Checklist.**
(DOC)
